# Open Reading Frame-3a gene of the 2019 novel coronavirus inhibits the occurrence and development of colorectal cancer

**DOI:** 10.1007/s12672-022-00473-6

**Published:** 2022-03-20

**Authors:** Han Shuwen, Wu Yinhang, Mao Jing, Chen Gong, Hou Xiaohui, Yang Xi, Wu Wei

**Affiliations:** 1 Department of Oncology, Huzhou Central Hospital, Affiliated Central Hospital Huzhou University, No. 1558, Sanhuan North Road, Wuxing District, Huzhou, 313000 Zhejiang China; 2 Graduate School of Second Clinical Medicine Faculty, Zhejiang Chinese Medical University, No. 548 Binwen Road, Binjiang District, Hangzhou, 310053 Zhejiang China; 3 Graduate School of Medical College of Zhejiang University, No. 268 Kaixuan RoadJianggan District, Hangzhou, 310029 Zhejiang China; 4 Clinical Medicine of Huzhou University, Medical College of Huzhou University, No. 759, Erhuan East Road, Huzhou, 313000 Zhejiang China; 5 Graduate School of Nursing, Huzhou University, No. 1 Bachelor Road, Wuxing District, Huzhou, 313000 Zhejiang China; 6 Department of Gastroenterology, Huzhou Central Hospital, Affiliated Central Hospital Huzhou University, No. 1558, Sanhuan North Road, Wuxing District, Huzhou, 313000 Zhejiang China

**Keywords:** 2019-nCoV, ORF3a, Colorectal cancer, Cell viability, Cell cycle, Apoptosis

## Abstract

Intestinal microecology is composed of bacteria, fungi and viruses. As a part of intestinal microecology, viruses participate in the occurrence and development of colorectal cancer. The 2019-nCoV was detected in stool samples from patients during COVID-19, suggesting that the 2019-nCoV may be associated with intestinal microecology. However, the relationship of the 2019-nCoV and CRC is unclear. The aim of this study is to explore the role of Open Reading Frame-3a (ORF3a) of the 2019-nCoV in CRC. After the pCDH-CMV-MCS-EF1-Puro vector that provides high expression of ORF3a was transfected into the SW480 CRC cell line, immunofluorescence was used to determine the localization of ORF3a in SW480 cells. The proliferation, migration, invasion, apoptosis, and cell cycle progression of SW480 cells were measured using the Cell Counting Kit-8 (CCK-8), wound healing, Transwell assay, flow cytometry, the TUNEL assay, and propidium iodide single staining. The results showed that ORF3a inhibited the proliferation, invasion, and migration of SW480 cells and induced their apoptosis after 24, 48, 72 h. Meanwhile, ORF3a inhibited the cell cycle and blocked SW480 CRC cells in the G1 phase. In in vivo experiments, high ORF3a expression was associated with decreased tumor volume, tumor weight, relative tumor volume, and tumor activity. ORF3a inhibited the growth and induced apoptosis and necrosis of tumor tissues. Based on this, we demonstrated that ORF3a might play a role in CRC, providing a new direction for the prevention and treatment of CRC.

**Supplementary Information:**

The online version contains supplementary material available at 10.1007/s12672-022-00473-6.

## Highlights

1. ORF3a was successfully transfected into SW480 cells, and its expression was localized in the cytoplasm of SW480 cells.
2. ORF3a inhibited SW480 cell proliferation, invasion and migration, induced their apoptosis, and blocked them in the G1 phase.
3. ORF3a inhibited colorectal tumor growth and accelerated cancer cell death in vivo.
4. Our study provides new insights into the use of viruses in anti-tumor regimens in colorectal cancer.

**Supplementary Information:**

The online version contains supplementary material available at 10.1007/s12672-022-00473-6.

## Introduction

Colorectal cancer (CRC) is the third most common malignancy worldwide and the second leading cause of cancer-related deaths [1]. An increasing amount of data indicate that intestinal flora imbalance may promote the occurrence of CRC [2]. The gut microenvironment is an extremely complex microecosystem. The diversity of organisms in the human intestinal tract is enormous. In addition to bacteria and fungi, hundreds of thousands of viruses are present [3]. In a recent study, researchers analyzed the gut metagenomes of 28,060 human samples and 2898 reference genomes of gut bacteria distributed worldwide. They constructed a database named “GDP” containing 142,000 sequences of intestinal phages [4]. Viruses can be directly involved in the occurrence of CRC by infecting cells or indirectly, by regulating the composition of the bacterial community [5]. Viral infections have been shown to induce cancer. The currently known viruses associated with CRC include human papillomavirus [6, 7], some herpes viruses, human immunodeficiency virus [8], and hepatitis B or hepatitis C virus (HCV) [9]. Studies have shown that cytomegalovirus herpesvirus infection is considerably associated with the incidence of CRC [7]. Hepatitis B virus infection not only induces liver cancer, but is also associated with liver metastasis in CRC [10]. However, there are few reports on the molecular mechanisms or specific effects of viruses on the occurrence and development of CRC.

Many coronavirus-related studies have been conducted owing to the novel coronavirus epidemic. The British Journal of Hematology published a special case in which a patient with Hodgkin's lymphoma was cured of cancer 4 months after being infected with the novel coronavirus [11]. Nonetheless, the exact mechanism by which the patient's tumor disappeared is unknown. Meanwhile, studies have shown that COVID-19 infections increase the risk of death in patients with cancer [12, 13]. Current studies have mostly focused on the effects of the virus on the outcomes of cancer; however, the understanding of the relationship between coronavirus infection and cancer development, metastasis, and sensitivity to treatment is still very limited. Fecal samples of COVID-19 patients were tested, and it was found that 2019-nCoV could still be detected in stool within 35 days after respiratory clearance [14]. It remains to be elucidated whether the 2019-nCoV, as an endogenous retrovirus, can act as an inducer or inhibitor for the occurrence of CRC, thereby affecting its course. By analyzing the genome sequence of COVID-19, we found that the novel coronavirus functional gene *ORF3a* might induce or inhibit CRC by acting on CRC driver genes.

The open reading frame (ORF) is a part of a gene sequence that encodes proteins and is involved in virus replication and release. The 2019-nCoV genome contains 14 ORFs encoding 27 proteins. The 3′ end contains four ORFs that encode structural proteins (S, E, M, and N) and eight ORFs that encode accessory proteins (ORF3a, 3b, P6, 7a, 7b, 8b, 9b, and ORF14) [15, 16]. ORF3a is a conservative coronavirus protein that contains different functional domains related to virulence, infectivity, and virus release [17]. ORF3a regulates viral mRNA translation and expression in the host [18, 19]. Although there are no reports on the relationship between ORF3a and tumor diseases, studies have shown that the ORF3a protein of 2019-nCoV 2 activates the NLRP3 inflammasome by promoting TNF receptor-associated factor 3 (TRAF3)-dependent ASC ubiquitination [20], and the activated NLRP3 inflammasome can improve colitis-related cancers induced by methoxymethane/dextran sodium sulfate in mice [21]. This suggests that the ORF3a protein may have an inhibitory effect on colitis-related cancers. Research has also shown that ORF3a of 2019-nCoV can induce cell apoptosis through exogenous pathways [22, 23].

Based on the above information, we transfected SW480 cells with the ORF3a cDNA using a lentiviral vector. In vivo and in vitro experiments were performed and pathological tissue analysis was conducted to examine the function of ORF3a in SW480 cell viability and apoptosis and characterize the role of ORF3a in the growth of colorectal tumors. The results of our present study are expected to provide a new research idea for the anti-tumor therapy of CRC using viruses.

## Materials and methods

### Lentivirus transfection

#### Construction of overexpression vector

We declare that all methods were performed in accordance with the relevant guidelines and regulations. The 2019-nCoV *ORF3a* gene was inserted into the expression vector pCDH-CMV-MCS-EF1-PURO vector (ORF3a overexpression vector). The vector pCDH-CMV-MCS-EF1-PURO was double digested with XbaI and BamHI at 37 ℃ for 2 h. Then, the products of enzyme digestion were analyzed using 1% agarose gel electrophoresis, and photographed using a gel imager (980A, Shanghai Furi Technology, Shanghai, China). Next, the DNA fragment of interest was recovered according to the instructions of the agarose gel recovery kit. The DNA solution was then collected, followed by seamless cloning. The recovered vector and the synthesized DNA fragments were subjected to homologous recombination and incubated at 50 °C for 15 min. The recombined product was then used to transform STBl3 competent cells. Transformed bacteria were evenly spread on a plate containing ampicillin and incubated overnight for colony formation. Three single clones were picked and cultured in 4 mL LB medium overnight. Then, a centrifuge was used to obtain the plasmid solution. The extracted plasmid was digested at 37 °C for 2 h. Electrophoresis was performed for 30 min: the electrophoresis buffer was 1 × TAE, the agarose concentration was 1%, and the electrophoresis voltage was 130 V. Pictures were taken on a gel imager for restriction digestion identification. The plasmids with the correct sequencing were extracted in large quantities and stored at − 20 °C.

#### Screening for cells stably expressing ORF3a

Briefly, 293T cells were plated into a 60 mm dish at a density of 1.5 × 10^6^ cells/dish. The next morning, after the cells adhered to the plate, the lentivirus was packaged. The initial lentivirus solution was stored in a refrigerator at 4 °C and used on the same day. Then, SW480 cells (Jiangsu Kaiji Biotechnology Company, Ltd., Nanjing, China) were infected with the virus. Cells in the logarithmic phase were washed with PBS, trypsinized to obtain a suspension of single detached cells, and 3 mL of complete culture medium was added for neutralization. The cell suspension was collected and centrifuged at 300×*g* for 3 min. The supernatant was discarded, and the precipitated cells were resuspended 1 mL complete medium, counted, and plated in a 6-well plate at a density of 3 × 10^5^ cells/well, and 2 mL medium was added to each well. The next day, after the cells were attached to the plate, the medium in the wells was discarded and replaced with L-15 complete medium containing 1000 μL virus supernatant. After 48 h, the infection efficiency was observed under a fluorescence microscope (IX73, Olympus, USA). If there were uninfected cells, puromycin was added at a final concentration of 0.5 µg/mL (pre-experimentally determined) to select for infected cells. The cells were cultured in the presence of the drug until all the cells showed green fluorescence. Some cells were cryopreserved, and some cell precipitates were collected to verify overexpression.

#### Confirmation of ORF3a overexpression using qPCR

RNA extraction was carried out with 1 mL TRIzol (9109, TaKaRa, Kyoto, Japan). DEPC H_2_O was used as the control (blank) for the determination of RNA purity and RNA quantification. Two microliters of RNA solution were aliquoted in an enzyme label analyzer (Epoch, BioTek, Vermont, USA) to determine the concentration and quality of the sample. Complementary DNA (cDNA) was synthesized from the extracted RNA using a cDNA synthesis kit (Fermentas; Thermo Fisher Scientific, Inc.), according to the manufacturer's protocol. RT-PCR experiments (ABI 7900HT FAST, USA) were performed by using the Power SYBR Green PCR Master Mix (A25742, Thermo, Waltham, USA). The primers (5′-3′) used were as follows: ORF3a-hF: GCAACGATACCGATACAAGCC, ORF3a-hR: CCAGCAGCAACGAGCAAAA; GAPDH-hF: TGACAACTTTGGTATCGTGGAAGG, GAPDH-hR: AGGCAGGGATGATGTTCTGGAGAG.

#### Analysis of cell proliferation using the CCK-8 assay

The CCK-8 assay was used to detect changes in SW480 cell proliferation. The cells were prepared into a cell suspension of 5 × 10^4^ cells/mL, and 100 μL cell suspension was added to each well of a 96-well cell culture plate (Corning Incorporated 3599, State of New York, USA). Then, the cells were cultured in a 5% carbon dioxide incubator (3111, Thermo) at 37 ℃ for 24, 48, or 72 h. Next, 10 μL CCK-8 (KGA317, Jiangsu Kaiji Biotechnology Company, Ltd., Jiangsu, China) was added to the cells of each well, and the absorbance at λ = 450 nm was measured. The growth inhibition rate was calculated. The experiment was repeated three times, and the difference in inhibition rates was compared between the three groups.

### Details of the authentication of the cells

SW480 [SW480] (Procell CL-0223) were kindly provided by Procell Life & Technology Co.,Ltd. A proper amount of SW480[SW-480] cells (NO. PC174, 1 × 10^6^) was taken and DNA was extracted using Chele × 100. 21 CELLID System was used to amplify 20 STR loci and sex identification loci, and ABI3130 × 1 genetic analyzer was used to produce PCR. The results were analyzed using GeneMapper IDX software (Applied Biosystems) and compared with ATCC DSMZ, JCRB, Cellosaurus Datebases, etc.

### Analysis of ORF3a expression using immunofluorescence

The differential expression of ORF3a protein in CRC tissues was determined using immunofluorescence assay. Before dewaxing, the tissue sections were placed at 25 ℃ for 60 min or baked in an incubator at 60 ℃ for 20 min. The tissue sections were immersed in xylene for 10 min and soaked for 10 min after replacing xylene. The samples were soaked in anhydrous ethanol, 95% ethanol, and 70% ethanol for 5 min each and washed twice with PBS for 5 min each. Antigen repair and paraffin-embedded tissue microarray for paraformaldehyde fixation. The sodium citrate buffer solution (pH 6.0) was heated to approximately 95 °C in an electric furnace or water bath. The tissue sections were placed in buffer solution and heated for 10 min. Following cooling at room temperature, the sections were removed and washed three times with PBS (5 min each). Normal goat serum (5%) was dropped onto tissue sections and incubated at room temperature for 30 min, after which the excess liquid was discarded. Then, the primary antibody was added and incubated at room temperature for 1 h or at 4 ℃ about 12 h or at 37 ℃ for 1 h. The sections were washed three times with PBS (5 min each). Fluorescent-labeled secondary antibodies were added and incubated at room temperature for 1 h in the dark. The cells were washed three times with PBS (5 min each). DAPI solution was then added to the section for 10 min. The sections were washed and stained again as above and then washed three times with PBS (2 min each). The difference in protein levels in CRC was determined. The experiment was repeated three times.

### Analysis of cell invasion using the Transwell assay

A Transwell assay was used to detect changes in SW480 cell invasion. The SW480 cells were divided into three groups: ORF3a blank group, ORF3a control group, and ORF3a high expression group. Cells in the logarithmic growth phase were digested with trypsin and inoculated into a six-well plate. The next day, after the cells adhered to the plate, the serum was removed from the cells, and the cells were starved in incomplete medium for 24 h. Simultaneously, Matrigel matrix glue (356234, BD, USA) was placed at 4 ℃ about 12 h to melt, and the melted Matrigel glue was diluted twice with incomplete culture medium. Diluted Matrigel (30 μL) was added into the upper chamber of Transwell and incubated at 37 ℃ for 120 min. Matrigel was then polymerized into a gel. The cells were digested with 0.25% trypsin and collected, and their density was adjusted to 1 × 10^5^ cells/mL with incomplete medium. The cell suspension (100 µL) was added into the Transwell chamber (3422, Corning Incorporated), and 500 µL of medium containing 20% FBS was added to the lower chamber. The plate was placed in a 5% carbon dioxide incubator. After 24 h, the matrix glue and cells in the upper chamber were wiped with a cotton swab. The Transwell was removed, placed upside down, and dried. Then, 500 µL of 0.1% crystal violet solution (C3886, Sigma, Bellefonte, Pennsylvania, USA) was added to a 24-well plate. The chamber was placed in a plate, and the membrane was immersed in the dye and incubated at 37 ℃ for 30 min. After PBS washing, an inverted biological microscope (IX51, Olympus, Japan) was used for observation. Three visual fields were photographed (magnification 200×), and the cells were counted. The experiment was repeated three times, and the difference in the number of cells between the three groups was compared.

### Analysis of cell migration using wound healing assay

A wound healing assay was used to detect SW480 cell migration. The cells in the logarithmic growth phase were digested and inoculated into six-well plates. The next day, when the cell adhered to the plate and reached approximately 80% confluence, the corresponding lentivirus was added according to the group setting. The sterile pipette tip was used to scratch a line across a six-well plate. The floating cells were washed off with PBS; then, fresh culture medium was added, and the plates were placed in a 5% carbon dioxide incubator for further culture for 24 h. After culture, the cells were photographed (magnification 100×), and the migration distance of the cells was measured. The experiment was repeated three times, and the difference in the migration distance between the three groups of cells was compared.

### Analysis of cell apoptosis using the annexin V-FITC/Propidium Iodide assay

FITC Annexin V Propidium Iodide (PI) was used to detect apoptosis in SW480 cells. The cells of the three groups were resuspended in 500 μL binding buffer. Then, 5 μL Annexin V-FITC and 5 μL PI [Annexin V-FITC/PI Apoptosis Detection Kit (KGA105, Jiangsu Kaiji Biotechnology Company, Ltd., Jiangsu, China] were added and mixed for 5–15 min at room temperature in the dark. Flow cytometry (Becton–Dickinson FACS Calibur, New Jersey, USA) was used to detect apoptosis at 24, 48 and 72 h. The percentages of the UL, LL, LR, and UR regions were calculated. The apoptosis rate was calculated according to the following equation (%) = LR (%) + UR (%).

### Analysis of cell cycle using PI single staining assay

The changes in cell cycle were detected using PI single staining assay. The three groups of logarithmically growing cells were trypsin digested and inoculated into well plates. The distribution of cells in the various phases of the cell cycle was determined using a cell cycle detection kit (KGA511, Jiangsu Kaiji Biotechnology Company, Ltd., Jiangsu, China). When the cells adhered to the plate after 24 h, the corresponding drug-containing medium was added according to the group setting, and a blank control group was established. Before staining, the fixing solution was washed with PBS; then, 100 μL RNase A was added in a 37 ℃ water bath for 30 min, followed by the addition of 400 μL PI for staining. The cells were protected from light at 4 ℃ for 30 min. Finally, a flow cytometer (FACSCalibur, Becton–Dickinson, New Jersey, USA) was used to record the red fluorescence at an excitation wavelength of 488 nm. The experiment was repeated three times, and the differences in cell cycle between the three groups were compared.

### Establishment of CRC-bearing mouse model and ORF3a high expression animal model

#### Ethics statement

Animal experiment has passed the animal ethics audit of Zhejiang University Laboratory Animal Center. The ethics number is 20617.

The purpose of this experiment was to analyze the effect of ORF3a on the colorectal tumor volume. 12 nude mice were equally divided into a control group and an ORF3a high expression group. SW480 cell suspensions of each group were inoculated subcutaneously into the right axilla of nude mice at a concentration of 1 × 10^7^ cells/mL. The diameter of the transplanted tumor was measured with a Vernier caliper. After 21 days of inoculation, the tumor grew to 80–100 mm^3^. Tumor diameter was measured to observe the growth of subcutaneously transplanted tumors in nude mice. At the end of the experiment, the nude mice were sacrificed, and the tumor mass was removed and weighed. Tumor volume (TV), relative tumor volume (RTV), and the evaluation index of anti-tumor activity: tumor growth inhibition rate (%) were calculated according to Eqs. (1), (2), and (3) to analyze the effect of ORF3A on the TV of transplanted nude mice.

1 $$ {\text{TV}} = {1}/{2} \times {\text{a}} \times {\text{b}}^{{2}} , $$

where a and b represent the length and width, respectively.

2 $$ {\text{RTV }} = {\text{ Vt}}/{\text{V}}0, $$

where V0 is the tumor volume measured in caged administration (i.e. d0), and Vt is the tumor volume at each measurement.

3 $$ \begin{aligned}&{\text{Tumor growth inhibition rate }}\left( \% \right) \\&\quad = \left( {{\text{average tumor weight of the model group}} - {\text{average tumor weight of the administration group}}} \right)\\ & \qquad/{\text{average tumor weight of the model group }} \times { 1}00\% .\end{aligned} $$

### Hematoxylin–eosin staining

Hematoxylin–eosin (HE) staining was performed to analyze the effect of ORF3a on the morphology of CRC cells. First, paraffin sections were prepared, dewaxed, and hydrated according to the conventional method. The sections were soaked in xylene for 5 min, then soaked in xylene again for another 5 min. The slices were then soaked in anhydrous ethanol, 95% ethanol, 85% ethanol, and 70% ethanol for 5 min each. After washing with PBS, the sections were immersed in HE dye solution of the kit (KGA224, Jiangsu Kaiji Biotechnology Company, Ltd., Jiangsu, China) for 3–5 min in the dye cylinder, washed with water for approximately 30–60 s, and immersed in the dichromatic solution I in the hematoxylin–eosin dye solution kit for approximately 20 s. After washing, the slices were immersed in tricolor II for approximately 40 s. After washing, the slices were stained with reagent 4 of the kit for 2 min. The excess dye was washed with Reagent 5 Color Enhancing Solution, washed twice, dried, and sealed. A digital pathological scanner (BX43, Olympus, Japan) was used to scan the sections, observe the characteristics, including tumor cell morphology, degree of necrosis, interstitial blood vessels, bleeding, and inflammatory cell infiltration, in the control group and the ORF3a high expression group, and score the tumor.

### Analysis of apoptosis using the TUNEL assay

The TUNEL assay was used to detect and analyze the effect of ORF3a on apoptosis in CRC tissues. First, paraffin sections were prepared using the method described above. Next, 1% Triton-100 was added to the samples, and the samples were placed at 25 ℃ for 10 min and soaked in PBS for 3 min × 3 times. Then, 50 µL 1xProteinaseK was dropped onto each sample and incubated at 37 ℃ for 30 min. After that, the sections were washed with PBS for 3 min × 3 times. The samples were treated with 3% H_2_O_2_-methanol solution for 15 min and washed with PBS for 3 min × 3 times to inactivate the enzyme, and then 50 µL TDT enzyme reaction solution was added to each sample and wetted in the dark at 37 ℃ for 1 h. Next, the samples were soaked in PBS for 3 min × 3 times for the TUNEL reaction. The sections were incubated with 50 µL Streptavadin-HRP at 37 °C for 30 min in the dark and washed with PBS for 3 min × 3 times. Two drops of freshly prepared DAB solution were added to each sample for the DAB color reaction; the depth of dyeing was observed under a microscope, stopped immediately after dyeing, gently rinsed with tap water for 15 min, and the color reaction was terminated with distilled water. Next, the slices were placed in hematoxylin dye solution, stained for 10 min, washed with distilled water, placed in hydrochloric acid methanol solution, and immediately washed with distilled water. The slices were then soaked in 70, 85, and 95% ethanol for 5 min each and immersed in anhydrous ethanol for 5 min. After soaking in xylene for 10 min, xylene was replaced and soaked again for 10 min. Neutral gum was added to the sections covered with coverslips. Finally, a light microscope was used to observe and take photos of the three high-expression regions for preservation.

### Statistical analysis

The data are expressed as the mean ± SD. Statistically significant differences between two groups were analyzed using Student's *t*-test, and multiple comparisons were performed using one-way analysis of variance (ANOVA). SPSS software (version 13.0) was used for all statistical analyses. Statistical significance was accepted at P < 0.05.

## Results

### Transfection of ORF3a into SW480 cells

**Fig. 1:**
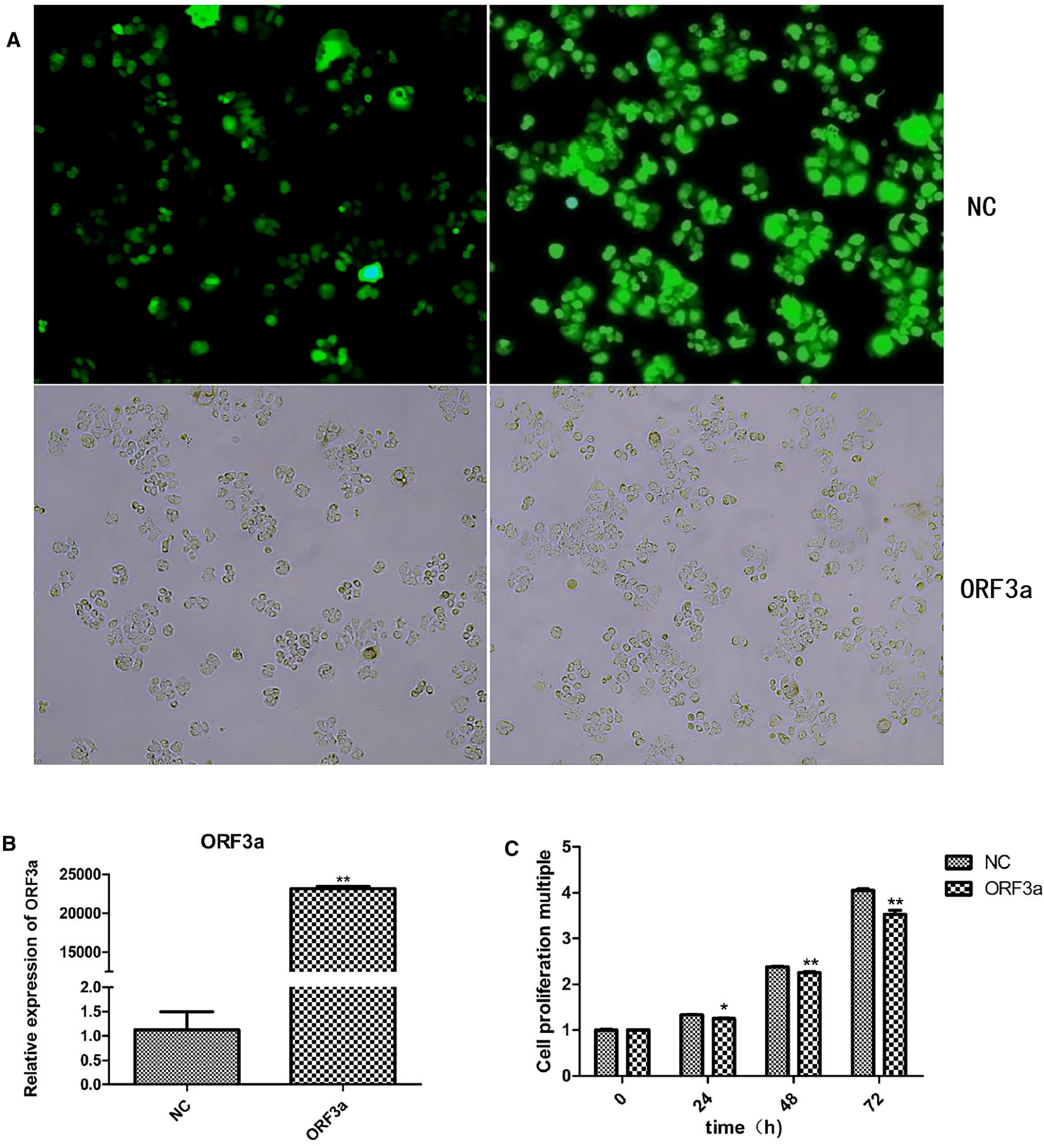
ORF3a was successfully expressed in SW480 cells. **A** Screening results for stable strain of the control virus. The control virus was packaged with a vector driving expression of GFP, and the overexpressing ORF3a vector did not drive the expression of GFP; hence, there was no fluorescence image. **B** qPCR results showing overexpression of ORF3a. **C** CCK8 assay results are presented as the mean ± SD values. Two-way ANOVA was used for statistical analysis; *P < 0.05 and **P < 0.01 were used as the screening criteria for significant differences and very significant differences, respectively

**Fig. 2:**
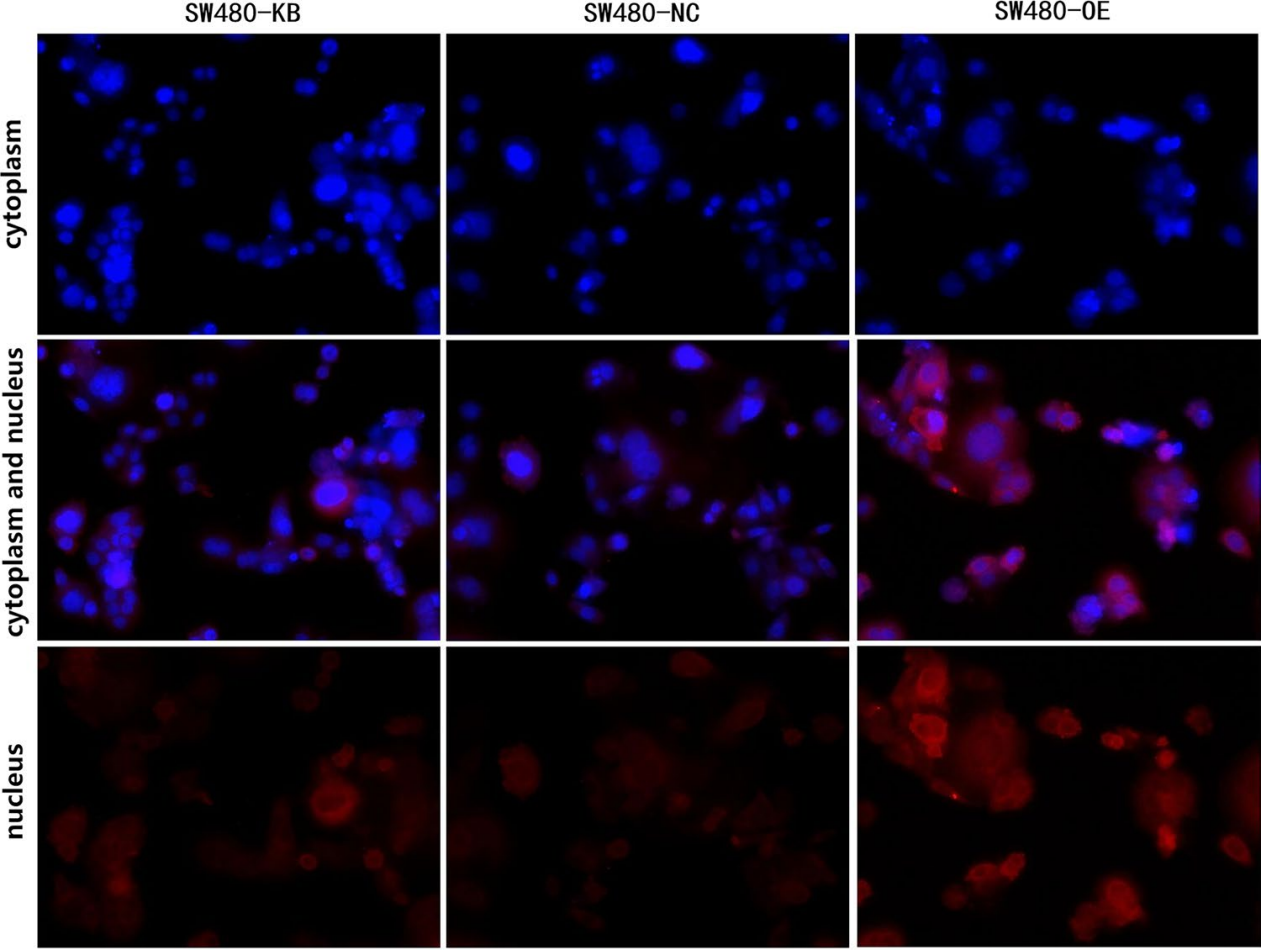
The localization of ORF3a in SW480 cells. The immunofluorescence was used to stain ORF3a protein in the blank, control, and ORF3a high expression groups. The first row represents protein staining in the cytoplasm (blue), the third row represents protein staining in the nucleus (red), and the middle row represents protein expression after the combination of cytoplasm and nucleus

To examine the role of ORF3a in CRC, we inserted the ORF3a target gene into the expression vector to establish the pCDH-CMV-MCS-EF1-Puro vector (ORF3a overexpression vector). After its introduction into SW480 cells by lentivirus infection, ORF3a was significantly overexpressed (P < 0.01), as measured using qPCR (Supplemental Figure 1). A cell model with high ORF3a expression was thus generated (Fig. 1). The overexpressed ORF3a protein was localized to the cytoplasm of SW480 cells (Fig. 2).

### ORF3a inhibits the viability and cell cycle of SW480 cells

**Fig. 3:**
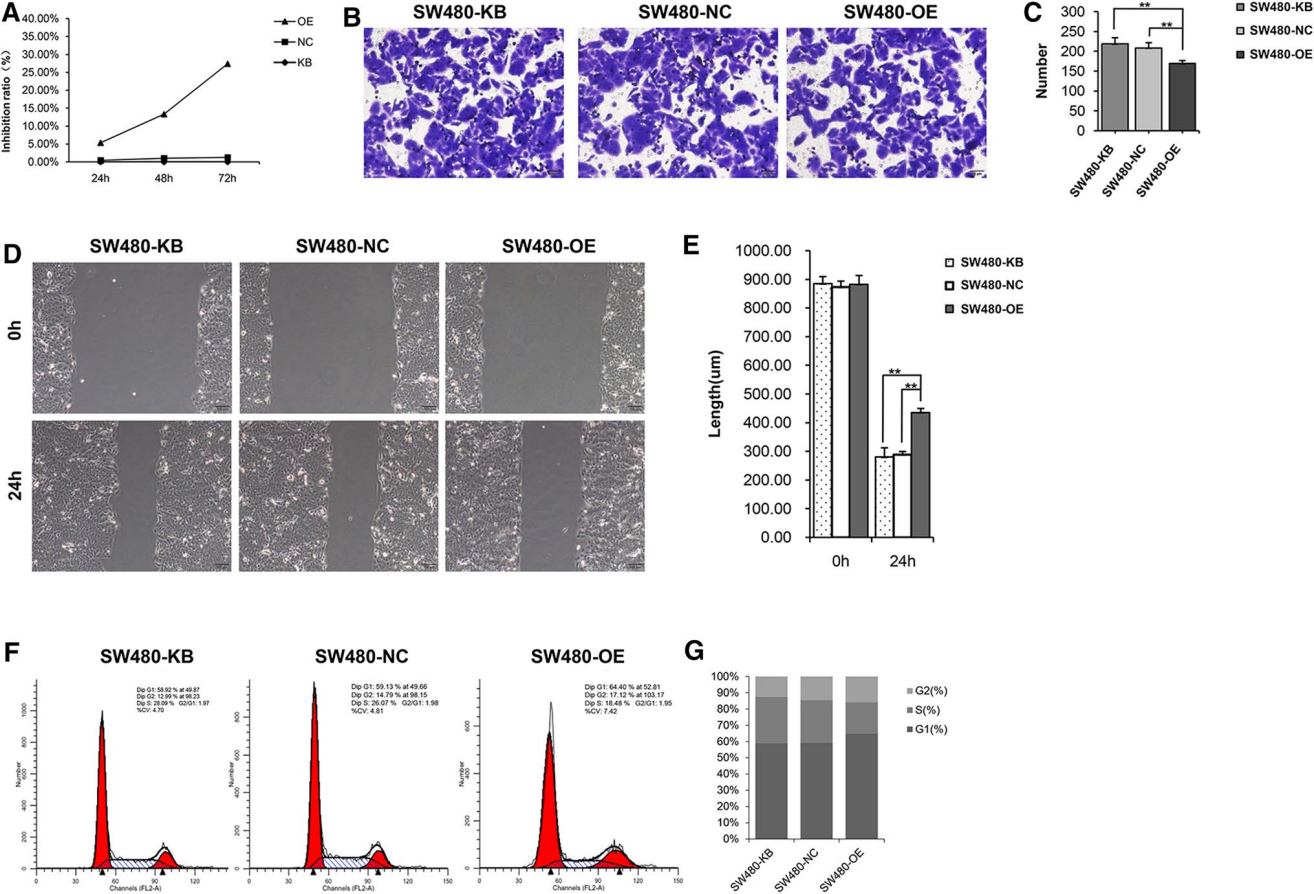
ORF3a inhibited cell viability and blocked cell cycle progression. **A** Cell proliferation of the blank group, control group, and ORF3a high expression group was compared. **B**, **C** Invasion of CRC cells. There was no significant difference between the blank and control groups (P > 0.05). There were statistically significant differences between the high expression group and the blank group and between the high expression group and the control group (**P < 0.001). **D**, **E** The migration length of cancer cells in the high expression ORF3a group was shorter (**P < 0.001), while the migration length of cancer cells in the blank group and the control group was longer (P > 0.05). **F**, **G** Cell cycle was detected using PI staining

ORF3a inhibited the proliferation of CRC cells. Moreover, the inhibitory effect of ORF3a on cell proliferation was enhanced with increase in time (Fig. 3A). Increased expression of ORF3a resulted in a sharp reduction in the number of cells migrating through the Transwell membrane, demonstrating the inhibitory effect of ORF3a on the invasion ability of CRC cells (Fig. 3B, C). ORF3a significantly inhibited the migration ability of CRC cells (Fig. 3D, E). Further research into the effect of ORF3a on the cell cycle of CRC found that ORF3a blocked SW480 CRC cells in the G1 phase (Fig. 3F, G).

### ORF3a inhibits the growth of tumor

**Fig. 4:**
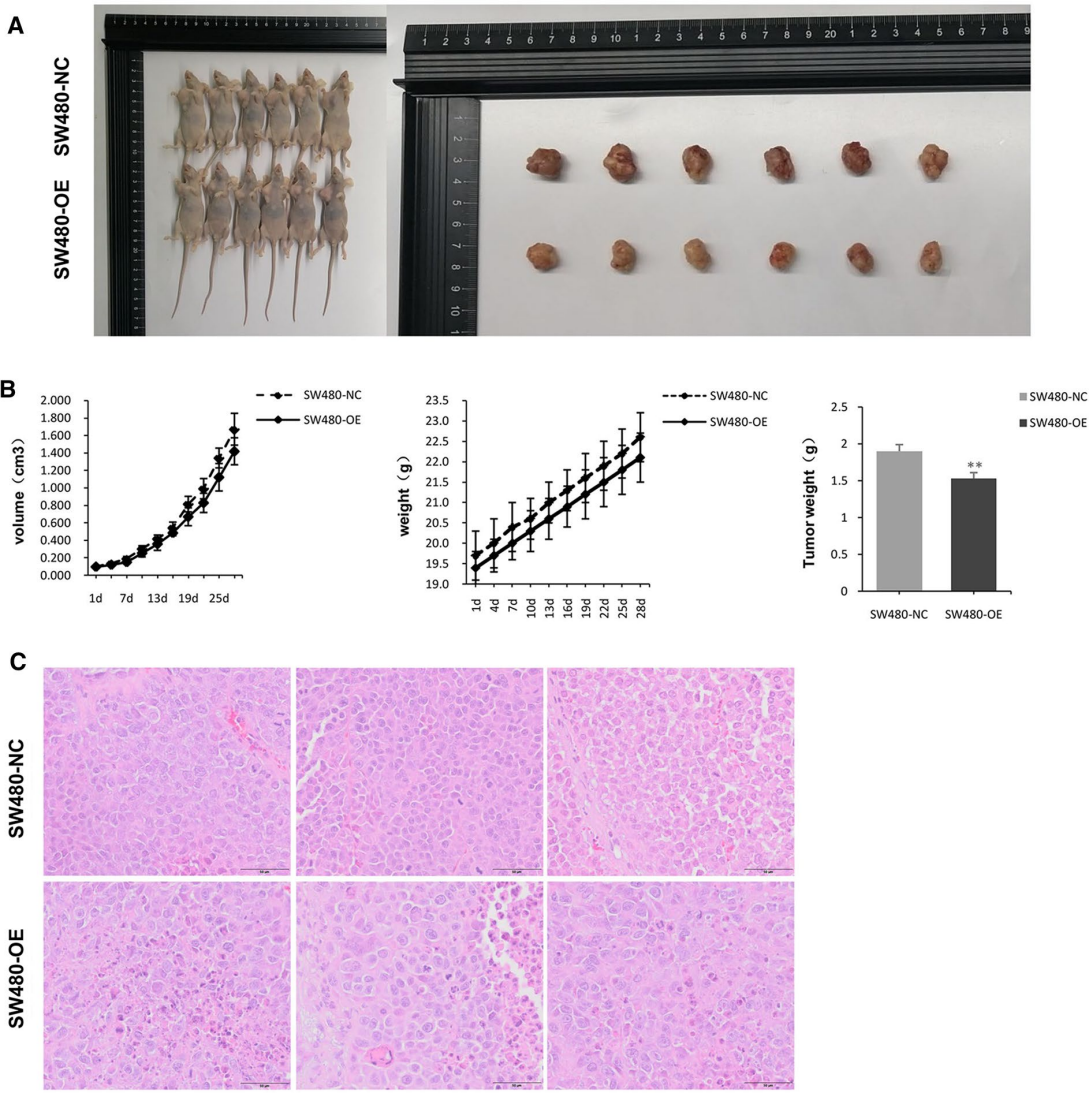
ORF3a inhibited tumor growth and promoted tumor death. **A**, **B** Comparison of tumor volume, body weight, and tumor weight between the control group and the ORF3a high expression group. **B** Comparison of tumor histological morphology between the control group and the ORF3a high expression group. **C** Comparison of tumor tissues between the control group and the ORF3a high expression group

The growth of subcutaneous tumors overexpressing ORF3a was examined in nude mice. ORF3a reduced the colorectal TV and weight (Fig. 4A, B). In the control group (NC), the tumor tissues had clear margins, compact texture, and no necrotic foci. No inflammatory cell infiltration or local hemorrhage was observed in the tumor tissues, and no neovascularization was observed in the stroma. In the ORF3a high expression group (OE), the margins of the tumor tissues were not clear, and focal necrosis was observed. No inflammatory cell infiltration or local hemorrhage was observed in the tumor tissues, and no neovascularization was observed in the stroma. ORF3a exerts anti-tumor effects by inducing apoptosis in CRC cells (Fig. 4C).

### ORF3a promotes apoptosis of SW480 cells

**Fig. 5:**
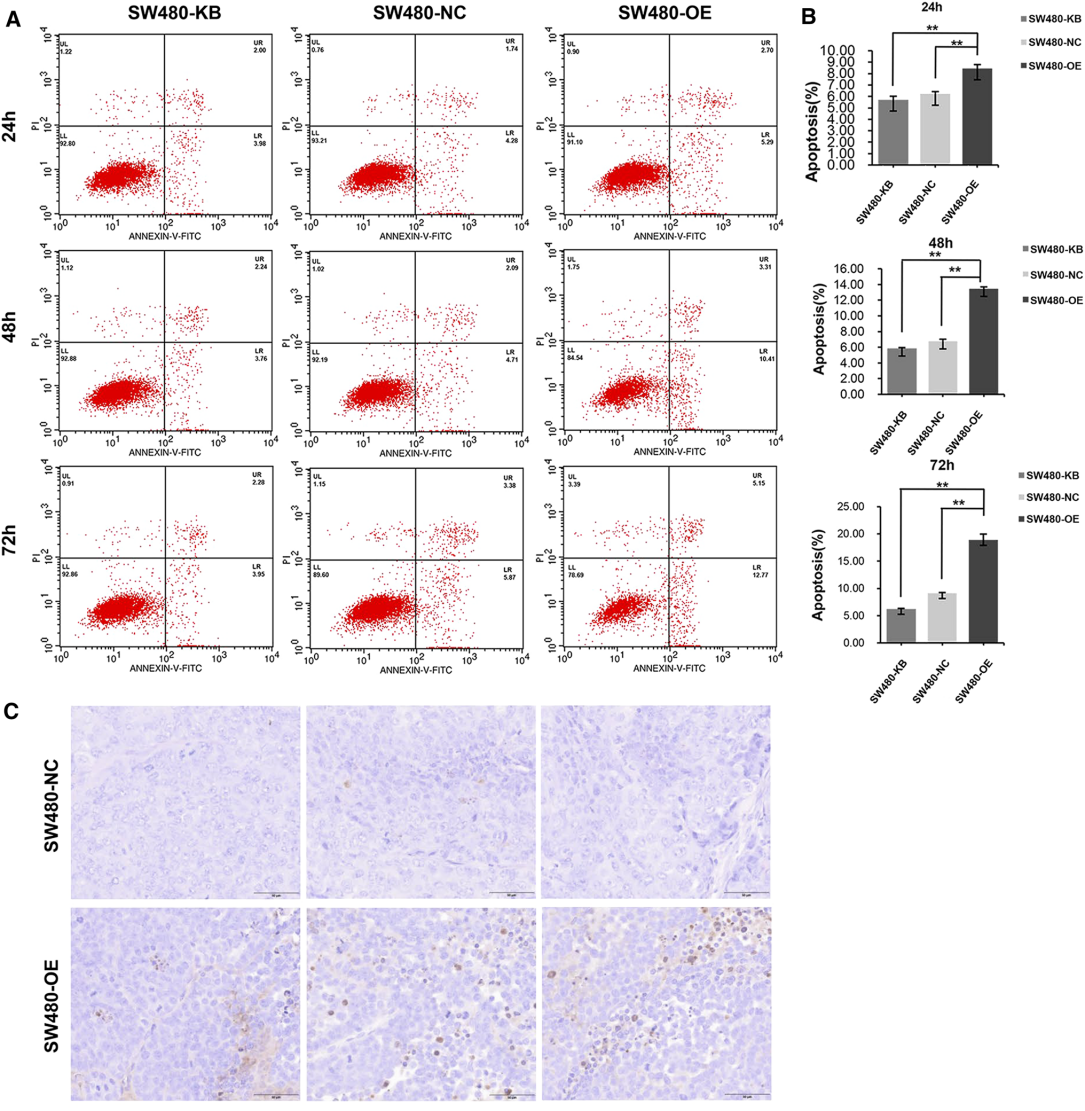
ORF3a induced apoptosis of CRC cells. **A**, **B** Apoptosis was detected by flow cytometry. The apoptosis rate in the high expression ORF3a group was higher than that in the blank and control groups (P < 0.001). There was no significant difference between the blank and control groups (P > 0.01). **C** Tissue staining of the mice showed that the apoptosis cells with high expression of ORF3a was more than that of the control group

The apoptosis of CRC cells was examined at 24, 48, and 72 h by using flow cytometry. The apoptosis rate of cells with high expression of ORF3a was higher than that of the blank and control groups (P < 0.001), and increased continuously as a function of time (Fig. 5A, B). In vivo experiments and pathological and morphological analyses of tumor tissues also revealed that ORF3a induced the apoptosis of tumor cells (Fig. 5C).

## Discussion

At present, COVID-19, characterized by severe acute respiratory syndrome, has developed into a global health threat [24, 25]. Viral infection is an important factor predisposing for cancer, and related reports have shown that coronaviruses are associated with certain tumors. It has been confirmed that the virus exists in the intestines of patients with COVID-19 [17, 26–28], and approximately 15% of patients with COVID-19 have gastrointestinal symptoms along with respiratory symptoms [29]. However, the relationship between coronavirus and CRC is not yet known. To explore the relationship between SARS-CoV-2 and CRC, we analyzed the RNA sequence of COVID-19 and found that its gene ORF3a might be a proto-oncogene that induces or inhibits CRC. Further study revealed that ORF3a of COVID-19 could act on CRC cells and have an impact on the occurrence, invasion, and migration of CRC.

First, we constructed a high-expression lentiviral vector of ORF3a of COVID-19 and successfully infected SW480 cells. Then, through in vitro cell experiments, we examined the effects of COVID-19 and ORF3a on the occurrence, invasion, migration, and apoptosis of CRC cells and on the differential expression of proteins in SW480 cells. Animal experiments and histopathological analysis confirmed that ORF3a can inhibit CRC tumor growth and induce apoptosis of CRC cells as well as tumor death.

Our study also verified that ORF3a inhibited SW480 cell cycle progression in vitro. ORF3a blocked SW480 cells in the G1 phase. All mammalian cells, including tumor cells, produce RNA and proteins rapidly during the period from mitosis to pre-DNA replication (G1 phase), and DNA replication in the next phase (S phase) is ready for material and energy. After the G1-S transition, cells undergo intense proliferation and DNA replication. As one of the most important stages of the cell cycle, the G1 phase is the limiting point for mammalian cell division and proliferation [30]. The G1 phase is characterized by complex and active molecular changes, which are easily affected by environmental conditions. In cancer cells, the control of the G1 phase limit point is lost for various reasons, resulting in uncontrolled proliferation, and the cells release a large number of wrong cell division signals, leading to the occurrence of cancer [31]. Therefore, ORF3a can block CRC cells at the G1 phase and inhibit their entry into the cell cycle and their division, thereby preventing proliferation, migration, and proliferation.

Ottaiano et al. [32] reported that (a) the tumor burden of three patients with metastatic colon cancer decreased unexpectedly following 2019-nCoV infection, (b) one patient with liver metastasis showed complete remission on CT scan 1 month after 2019-nCoV infection, and (c) two other patients infected with 2019-nCoV showed an unexpected decrease in their metastases one to three months later. These studies showed that 2019-nCoV has an inhibitory effect on colon cancer, and ORF3a may be the key responsible factor, which is consistent with our results. The specific mechanism of the correlation between 2019-nCoV and colon cancer needs to be further studied and verified.

In addition, studies have shown that patients with active 2019-nCoV gastrointestinal infections experience changes in their intestinal flora. After the 2019-nCoV respiratory tract infection recovery, even if there are no gastrointestinal symptoms, long-term "Latent" gastrointestinal infection signs will still be present [33]. This indicates that 2019-nCoV infection causes long-term effects and affects the gastrointestinal tract. Therefore, it is necessary to study the relationship between 2019-nCoV and CRC, and ORF3a was proven to be a very good starting point.

The effects of ORF3a on CRC are first reported. The expression of virus or non-virus associated ORF protein has been reported to be related to tumor. Overexpression of ORF48 on chromosome 8 can reduce the proliferation, migration and invasion of CRC cells, and play an inhibitory role in CRC by inhibiting MAPK signaling pathway [34]. Alternative T-Antigens, a tumor-associated antigen encoded by ORF5 in human polyomavirus, is thought to disrupt the signaling pathway of tumor cell proliferation [35]. When expressed alone, adenovirus ORF4 protein induces an evolutionarily conserved, Caspase-independent, cancer-selective cell death [36].

Although the role of COVID-19 infection and its functional gene ORF3a in the occurrence and migration of CRC was partially clarified through cell and animal experiments, further studies on the molecular mechanism are still needed. The molecular pathway and the factors involved in the inhibitory effect of ORF3a on CRC need to be clarified. At the same time, under effective and strict epidemic prevention and control policies, the number of patients infected with COVID-19 complicated with CRC is very small, and it is difficult to carry out clinical validation studies. The changes in the prevalence of CRC, tumor progression, lung metastases from CRC, and treatment sensitivity among patients with COVID-19 should be evaluated. In addition, the results of this study were obtained on a single cell line and animal model, so it is necessary to verify the results through multiple cell lines or animal models. Elucidation of the specific function of ORF3a in CRC is expected to provide a scientific basis for a new anti-tumor strategy for CRC.

## Conclusion

In the present study, we demonstrated for the first time the inhibitory effect of the novel coronavirus ORF3a on the influence of CRC. Compared with CRC without ORF3a expression, cell viability, cell division and replication, and tumor growth were inhibited when ORF3a was overexpressed. Moreover, the expression of nuclear and cytoplasmic proteins in CRC was affected by ORF3a. These findings can contribute to a better understanding of the mechanism through which the virus affects the occurrence and progression of CRC and provide a new direction for the treatment of CRC.

## Supplementary Information

Below is the link to the electronic supplementary material.

Supplementary file1 (TIF 54,373 KB). Vector map of overexpressed ORF3a and electrophoresis of overexpressed plasmid. A: The ORF3a target gene was inserted into the expression vector to establish the pCDH-CMV-MCS-EF1-Puro vector (ORF3a overexpression vector). B: After its introduction into SW480 cells by lentivirus infection, electrophoresis of the plasmid suggested ORF3a was significantly overexpressed (P < 0.01)

Supplementary file2. (DOCX 15 KB)

## Data Availability

The datasets generated during the current study are not publicly available but obtained from corresponding authors on reasonable request.
