# Supplementary figures and images for: Open Reading Frame-3a gene of the 2019 novel coronavirus inhibits the occurrence and development of colorectal cancer
Source: Discov Oncol. 2022 Mar 20;13:14. doi: 10.1007/s12672-022-00473-6 (PMC8934246; doi:10.1007/s12672-022-00473-6)

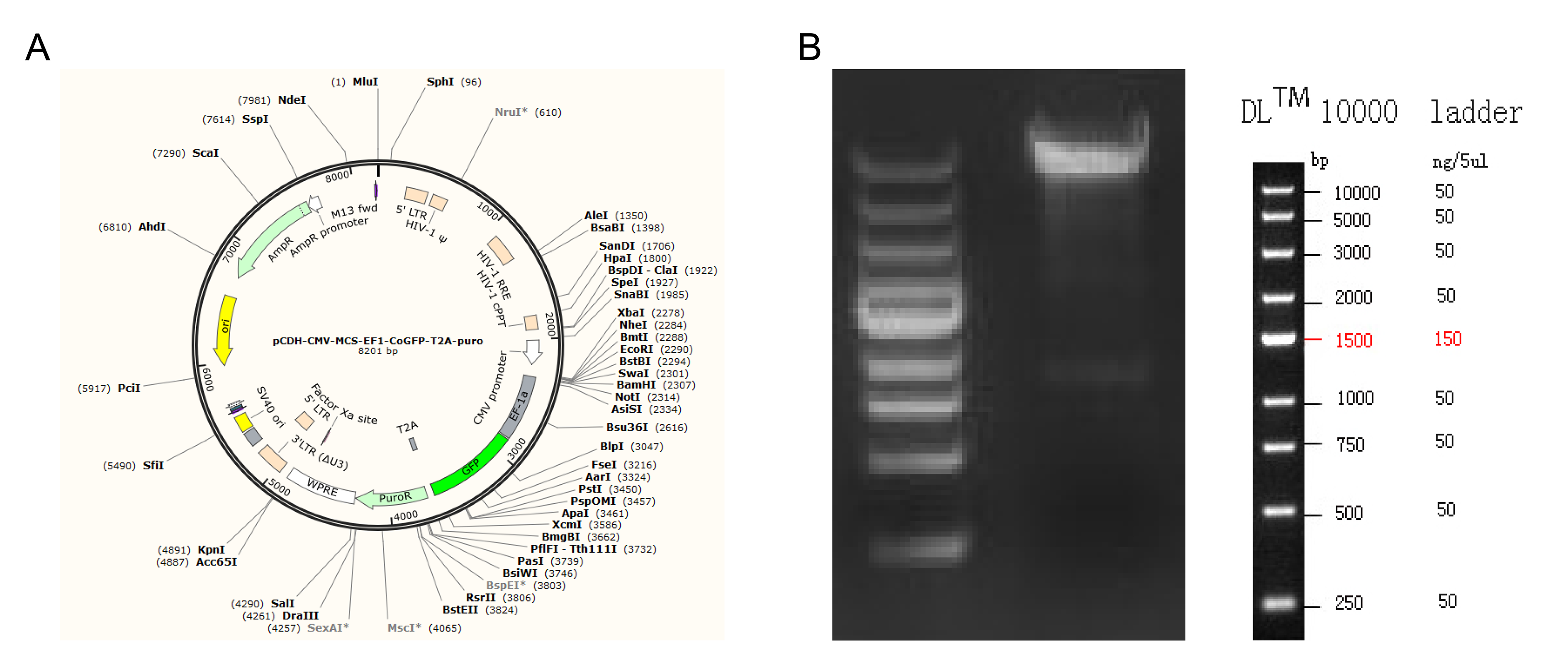

Supplement: Supplementary file 1 — Supplementary file1 (TIF 54,373 KB). Vector map of overexpressed ORF3a and electrophoresis of overexpressed plasmid. A: The ORF3a target gene was inserted into the expression vector to establish the pCDH-CMV-MCS-EF1-Puro vector (ORF3a overexpression vector). B: After its introduction into SW480 cells by lentivirus infection, electrophoresis of the plasmid suggested ORF3a was significantly overexpressed (P < 0.01) [file 12672_2022_473_MOESM1_ESM.tif]
